# Three-Dimensional Quantitative Morphometric Analysis (QMA) for *In Situ* Joint and Tissue Assessment of Osteoarthritis in a Preclinical Rabbit Disease Model

**DOI:** 10.1371/journal.pone.0147564

**Published:** 2016-01-25

**Authors:** Kathryn S. Stok, Bryce A. Besler, Thomas H. Steiner, Ana V. Villarreal Escudero, Martin A. Zulliger, Markus Wilke, Kailash Atal, Aurelie Quintin, Bruno Koller, Ralph Müller, Dobrila Nesic

**Affiliations:** 1 Institute for Biomechanics, ETH Zurich, Zurich, Switzerland; 2 SCANCO Medical AG, Bruttisellen, Switzerland; 3 Department of Clinical Research, University of Bern, Bern, Switzerland; INSERM - university Paris 7, FRANCE

## Abstract

This work utilises advances in multi-tissue imaging, and incorporates new metrics which define *in situ* joint changes and individual tissue changes in osteoarthritis (OA). The aims are to (1) demonstrate a protocol for processing intact animal joints for microCT to visualise relevant joint, bone and cartilage structures for understanding OA in a preclinical rabbit model, and (2) introduce a comprehensive three-dimensional (3D) quantitative morphometric analysis (QMA), including an assessment of reproducibility. Sixteen rabbit joints with and without transection of the anterior cruciate ligament were scanned with microCT and contrast agents, and processed for histology. Semi-quantitative evaluation was performed on matching two-dimensional (2D) histology and microCT images. Subsequently, 3D QMA was performed; including measures of cartilage, subchondral cortical and epiphyseal bone, and novel tibio-femoral joint metrics. Reproducibility of the QMA was tested on seven additional joints. A significant correlation was observed in cartilage thickness from matching histology-microCT pairs. The lateral compartment of operated joints had larger joint space width, thicker femoral cartilage and reduced bone volume, while osteophytes could be detected quantitatively. Measures between the *in situ* tibia and femur indicated an altered loading scenario. High measurement reproducibility was observed for all new parameters; with ICC ranging from 0.754 to 0.998. In conclusion, this study provides a novel 3D QMA to quantify macro and micro tissue measures in the joint of a rabbit OA model. New metrics were established consisting of: an angle to quantitatively measure osteophytes (σ), an angle to indicate erosion between the lateral and medial femoral condyles (ρ), a vector defining altered angulation (λ, α, β, γ) and a twist angle (τ) measuring instability and tissue degeneration between the femur and tibia, a length measure of joint space width (JSW), and a slope and intercept (m, Χ) of joint contact to demonstrate altered loading with disease progression, as well as traditional bone and cartilage and histo-morphometry measures. We demonstrate correlation of microCT and histology, sensitive discrimination of OA change and robust reproducibility.

## Introduction

Recently, a panel of experts, together with the US Food and Drug Administration described osteoarthritis (OA) as a complex, progressing and multiscale disease [1] affecting not only articular cartilage but also subchondral bone, ligaments, menisci, surrounding muscles and synovium. Structural deterioration of joint tissues leads to muscle atrophy, limb deformity and eventually loss of function [1, 2]. Understanding disease development and subsequent establishment of efficacious treatment strategies have been confounded by the inability to visualise the condition of the cartilage and quantitatively assess and monitor pathological changes.

Technological advances have led to a number of approaches to image joints for preclinical and clinical research purposes. Animal OA models represent an important element in the quest to understand, monitor and prevent disease development, as well as effectively evaluate the arrest of progression with treatment [3]. The advancement of disease is usually linked with progressive deterioration of cartilage, thus non-invasive approaches to assess cartilage damage have been considered crucial for development of novel therapies.

Clinically, magnetic resonance imaging (MRI) allows identification of morphological changes in damaged cartilage [4], including determination of cartilage thickness and volume [5]. Furthermore, MRI parametric mapping techniques, such as cartilage transverse relaxation time (T2), can distinguish biophysical properties of the tissue [6], while contrast-enhanced techniques, such as dGEMRIC, have demonstrated success in monitoring proteoglycan content both *in vitro* and clinically [7, 8]. These methods have been translated into small and medium-sized preclinical animal models, but suffer from resolution restrictions of MRI in visualising cartilage (usually 1–2 pixels for full thickness) [9–12].

Micro-computed tomography (microCT) is rapid, accurate and offers necessary spatial resolution to visualise and quantify morphology in small animal joints. Until recently, however, it has been limited to bone tissue imaging due to weak attenuation of cartilage in radiography. Cartilage visualisation has been improved in microCT with various contrast agents or phase-contrast for *ex vivo* evaluation [13–19]. The basic method for contrast-enhanced CT involves bulk staining of cartilage with a radiographic contrast agent for *ex vivo* imaging of cartilage and quantification of proteoglycans, i.e. Hexabrix^®^ (ioxaglate meglumine 39.3%, ioxaglate sodium 19.6%), as originally described by Palmer *et al* [17]. Various contrast options offer advantages depending on whether the models target early- or late-stage OA [20, 21]. Very few *in situ* (i.e. articulated) imaging studies have been performed using these methods (contrast CT [22] or phase-contrast CT [18]), but they presented difficulties when delineating cartilage boundaries *in vivo*. Other contrast agents, which are injected into the joint space to create a negative contrast of the cartilage volume, do not permeate the soft tissues and allow boundary delineation [23] However, these contrast agents can be quite expensive and/or toxic. SiO_2_-micro beads have been shown to give good boundary delineation [24] and are a cheap alternative for exploratory studies. Independently of the contrast agent used, 3D quantification has remained restricted to cartilage thickness and volume [18, 25–27].

MicroCT provides 3D image datasets that can be exploited to access 3D metrics that define preclinical OA in various animal models. In this work, we aim to utilise advances in multi-tissue imaging, and cast the net wider to incorporate metrics which define *in situ* joint changes alongside individual tissue changes. We demonstrate a protocol for processing intact animal joints for microCT; both dissection and staining of tissues, and microCT settings to visualise relevant joint, bone and cartilage structures for evaluating arthritis in a preclinical rabbit model. The rabbit ACLT is chosen as it is surgically suitable, leads to rapid onset of disease, and is representative of a medium-sized OA animal model [3]. Furthermore, structural alterations to the cartilage tissue for this model are well-described in literature [28]. The methodology is assessed against the gold standard—histology—and a correlation between the methods is established. Furthermore, a comprehensive 3D quantitative morphometric analysis (QMA) of macroscopic and microscopic changes in the knee joint of a preclinical rabbit OA trauma model is performed, including analysis of reproducibility of the quantitative measures. Evaluated metrics include an angle to quantitatively measure osteophytes (σ), an angle to indicate erosion between the lateral and medial femoral condyles (ρ), a vector defining altered angulation (λ, α, β, γ) and a twist angle (τ) measuring instability and tissue degeneration between the femur and tibia, a length measure of joint space width (JSW), and a slope and intercept (m, Χ) of joint contact to demonstrate altered loading with disease progression, as well as traditional bone and cartilage and histo-morphometry measures.

## Materials & Methods

**Fig 1:**
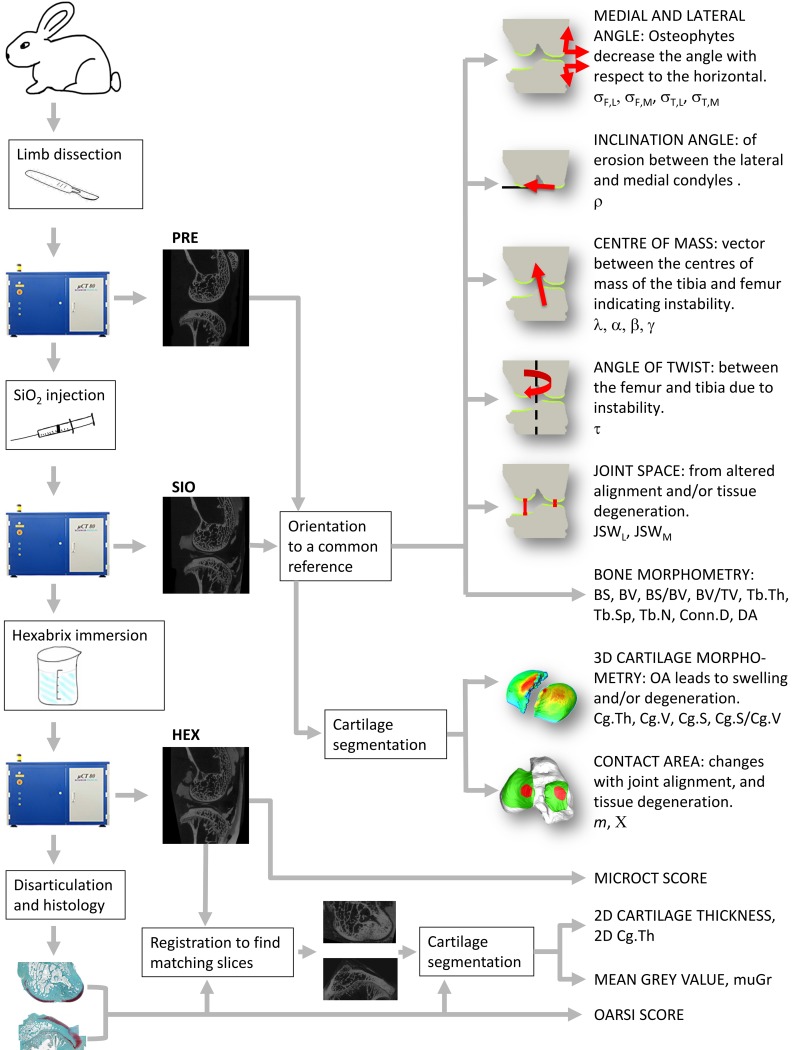
Overview Schema. Schema illustrating key aspects of the experimental procedure and parameters of interest.

A schema illustrating key aspects of the study design and parameters of interest is shown in Fig 1.

### Animals and anterior cruciate ligament transection surgery

The study protocol was approved by the Cantonal Ethics Commission of Bern (Permit Number: 49/10). For the OA trauma model, eight healthy female New Zealand white rabbits aged 4.5 months weighing 3.5 ± 0.4 kg underwent anterior cruciate ligament transection (ACLT) on the right knee (OP) [3, 29]. The contralateral, left joint served as a control (NO). Premedication consisted of a combination of Ketamine (65 mg/kg, Narketan 100 mg/ml, Vétoquinol) and Xylazine (4 mg/kg, Xylapan 20 mg/ml) given intramuscularly. General anesthesia was induced via infusion of the same concentration of Ketamine and Xylazine for the duration of surgery. Post-operative treatment comprised analgesia (Buprenorphin, Temgesic, 0.3 ml/rabbit at 0.3 mg/ml) and antibiosis (0.5 ml Duplocillin, 150,000 IU) after surgery and once for the 2 following days. Animals were sacrificed 8 weeks post-operatively: immobilized rabbits received an intravenous injection of an overdose of Pentobarbitol. The intact joints were dissected to include soft tissue, and stored at 4°C until scanning (max 3 hours).

For reproducibility testing, the tibio-femoral joints of seven age-matched, New Zealand white rabbits (waste material donated from an unrelated study) were obtained as described above.

### MicroCT scan protocol

MicroCT scans (SCANCO Medical AG, Brüttisellen, Switzerland) were performed with a 300 ms integration time, a 70 kVp source voltage, 8 W power, and an isotropic voxel size of 18 μm. The volume of interest included the femoral condyles and epiphyseal bone as an upper limit, and the tibial plateau and epiphyseal bone as a lower limit (approximately 35–40 mm). A consistent positioning during scan preparation was achieved with a wedge (with an angle of 160°) placed behind the knee to control flexion-extension. For the OA study, three scans were made per joint: PRE, SIO and HEX. Excess muscle and fat surrounding the joint was removed (while ensuring the joint capsule stayed intact) and samples were scanned (PRE) as described above. Afterwards, samples were flexed to loosen the joint. A contrast solution of 25 ml of SiO_2_-micro beads (0–20 μm diameter) (SWARCO Vestglass GmbH, Recklinghausen, Germany) was mixed with 30 ml phosphate buffered saline (PBS), centrifuged for 15 minutes to remove air bubbles, and then gently stirred. Five ml of the resulting solution was injected into the joint space in 3–5 doses to fill the joint cavity. The joint was flexed and massaged for 1 min between doses to ensure even contrast distribution around and between the soft tissues. The contrast agent was unable to penetrate the soft tissues. The samples were scanned again (SIO) and subsequently placed in a 60% PBS/40% Hexabrix^®^ (Mallinckrodt, Hazelwood, USA) solution for 24 hrs at 4°C. Samples were rinsed with PBS, scanned (HEX), and left in PBS at 4°C for histology (max 24h).

For reproducibility testing, the same protocol was applied on seven additional rabbit joints, with two additional HEX scans, including re-positioning between scans (HEX1, HEX2, HEX3).

### Histological analysis

Joints (n = 16) were disarticulated, and femurs and tibias decalcified, dehydrated, and embedded in paraffin. Serial sagittal 3 μm sections comprising cartilage and underlying bone were stained with Safranin-O/Fast Green (Fluka, Sigma Aldrich, St Louis, MO, USA). Three histology sections per femur and tibia (n = 96) were digitised using a light microscope (Leica DM/RB, Leica AG, Germany) and stitching a series of overlapping sub-images using ImageJ [30].

### 2D image analysis

Each histology image was registered within the HEX scan using a custom C++ script [31]. The cartilage was manually segmented for each matching histology and HEX image (Photoshop CS5, Adobe Systems, CA, USA) and mean 2D cartilage thickness (2D Cg.Th [μm]) was measured [32]. Mean greyscale (muGr [–]) was calculated for the histological cartilage using a custom filter that converts Safranin-O/Fast Green stain to greyscale [33], and for the matching microCT slice. Three users segmented a random subselection of 10 femur and 10 tibia image pairs from both NO and OP joints to demonstrate user independence of the segmentation procedure.

### Histological and microCT scoring

Semi-quantitative histological evaluation was performed on Safranin-O/Fast Green stained slides (n = 96) using OARSI histopathology guidelines [28]. OARSI score was based on: Safranin-O stain (0–6), tissue structure (0–11), chondrocyte density (0–4) and cluster formation (0–3). Two additional parameters, osteophytes (0–4) and tidemark breach (0–3), were included to obtain a total histology score. MicroCT score, performed on matching HEX images (n = 96), was based on: surface structure (0–3), cartilage thickness (0–3), osteophyte size (0–4), change in osteophyte grey shade relative to normal bone, (0–1), and evidence of bone remodelling (0–1).

### 3D quantitative morphometric analysis (QMA)

All microCT data were reconstructed to a common axial angle in the transverse plane, and filtered using a constrained 3D Gauss filter (σ = 1.2, s = 1). Cartilage thickness (3D Cg.Th [mm]), volume (Cg.V [mm^3^]), surface (Cg.S [mm^2^]), and surface-to-volume ratio (Cg.S/Cg.V [mm^-1^]) were calculated from the segmented scans [27].

To detect osteophytes, all joints were registered and aligned to a reference femoral orientation—where the long axis of the reference femur was aligned with the vertical z-axis—using a B-spline interpolation to reduce rotational errors [34]. The local minima in the lateral and medial condyles and local maxima in the condylar notch were located by the algorithm, and the average inclination of the lateral, σ_F.L_ [°], and medial condyle, σ_F.M_ [°] were calculated relative to these maxima and minima. Additionally, to detect erosion between the lateral and medial femoral condyles, the average inclination of the straight line joining the local minima, ρ[°], was also calculated. The same process was repeated for reference tibial orientation (where the long axis of the reference tibia was aligned with the z-axis) to calculate average inclination of the lateral, σ_T.L_ [°], and medial plateau, σ_T.M_ [°].

Bone morphometry of cortical and epiphyseal trabecular bone of the tibia and femur was performed as previously described [35]. Measurements of cortical thickness, Ct.Th [mm]; porosity, Ct.Po [%]; bone volume fraction, BV/TV [%]; surface, BS [mm^2^]; volume, BV [mm^3^], surface-to-volume ratio, BS/BV [mm^-1^]; trabecular thickness, Tb.Th [mm]; spacing, Tb.Sp [mm]; number, Tb.N [mm^-1^]; connectivity density, Conn.D [mm^-3^]; and degree of anisotropy, DA [1] were performed.

The reference tibial orientation—where the long axis of the tibia was aligned with the vertical z-axis—was used to quantify *in situ* joint morphometry. Landmarks for calculating JSW were found by averaging the centre of geometry of the 100 most distal slices of the femur in the transverse (XY)-plane, and then shifting this coordinate on to the most distal femoral slice to locate the minima on each of the lateral and medial condyles. This technique was used to further minimise positional errors. JSW was taken from this landmark to the point of first contact with the tibial surface resulting in JSW_L_ [mm] and JSW_M_ [mm], respectively. To investigate potential tibio-femoral shift with disease, a vector with length, λ [mm], and orientation, α [°], β [°], γ [°], was defined as the centre of mass of the femur relative to the centre of mass of the tibia along the three principle axes in a Cartesian coordinate system (coronal view along YZ and sagittal view along XZ plane). Additionally, as a surrogate measure of altered twist between the femur and tibia, the difference in yaw angle, τ [°], needed to align the femur and tibia to their respective common orientations, was calculated. Finally, to measure differences in tibio-femoral contact due to induced OA, the contact area between the femur and tibia—including cartilage volumes—was calculated when virtually loading the femur onto the tibia in a stepwise manner. At each step (voxel dimension), contact area was calculated and plotted against step size, and distance travelled to first contact for the medial and lateral aspects, χ [μm], and rate at which contact area increases, *m* [mm^2^/mm], were obtained.

### Statistics

Statistical analysis was performed using SPSS (20.0, IBM, NY, USA). All data were tested for normality using the Shapiro-Wilk test. Pearson correlation coefficient, R, was used to compare histology and microCT images for measurement of 2D Cg.Th and muGr (p < 0.05). Bland-Altman plots were drawn to check for proportional bias between the two methods. In order to assess user independence of the segmentation procedure, a two-way mixed model intraclass correlation coefficient (ICC) with a 95% confidence interval (CI) was calculated (i.e. to test absolute agreement for single measurements) [36, 37].

To test the sensitivity of QMA for detecting differences between operated (OP) and contralateral (NO) joints, a linear mixed-effects model with post-hoc Bonferroni adjustment to correct for multiple comparisons was used to test for significant differences (p < 0.05). Individual rabbits were considered random effects, and—where appropriate—medial/lateral and tibia/femur were treated as within-sample, fixed effects. Interaction between site and bone of interest was investigated. Additionally, bivariate linear regression analyses were used to evaluate associations between 3D QMA variables (bone, cartilage, and joint associations), as well as scores from microCT and histology (p < 0.05). The correlation coefficient, R, and coefficient of determination R^2^, ascertaining the goodness-of-fit of the model, were computed along with the fit coefficients of the model; p < 0.05.

Reproducibility of 3D QMA was tested with a two-way mixed model intraclass correlation coefficient (ICC) with a 95% confidence interval (CI) for all variables. Additionally, precision errors were calculated and expressed as both, absolute error, PE(SD) and percentage of coefficient of variation, PE(%CV) of repeated measurements [38].

## Results

### 2D image analysis

**Fig 2:**
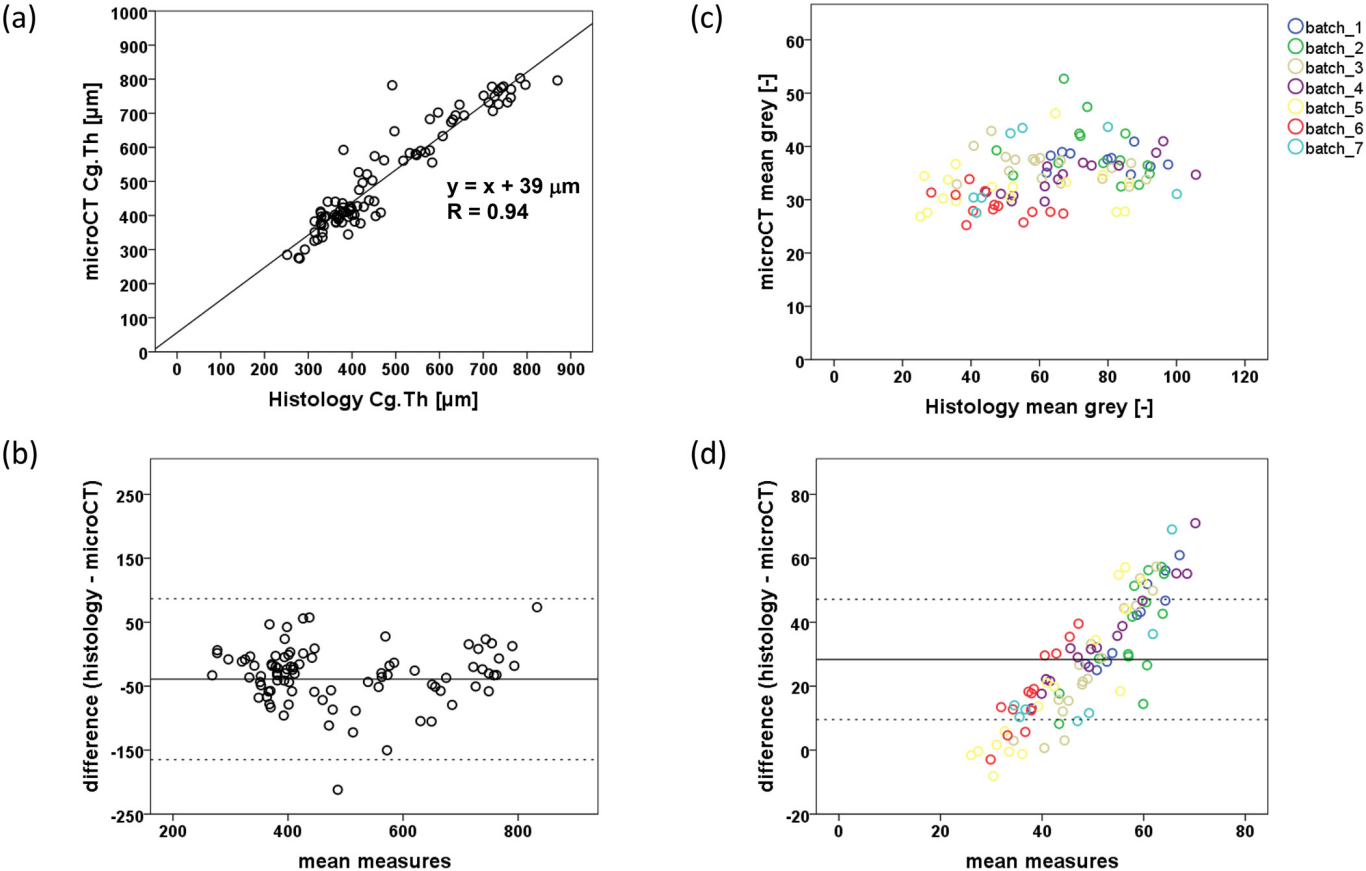
Comparison of microCT and histology. (a) Plot of 2D cartilage thickness measured by microCT against histology, R = 0.94, p < 0.001, and (b) a Bland-Altman plot showing relatively even scatter at both low and high values of mean measures, indicating no obvious trend of increasing scatter with Cg.Th. (c) Plot of mean grey value measured with microCT against histology (grouped by staining batch), and (d) a Bland-Altman plot showing the spread of scatter points with a strong proportional bias with increasing mean measures, independent of histology staining batch.

**Fig 3:**
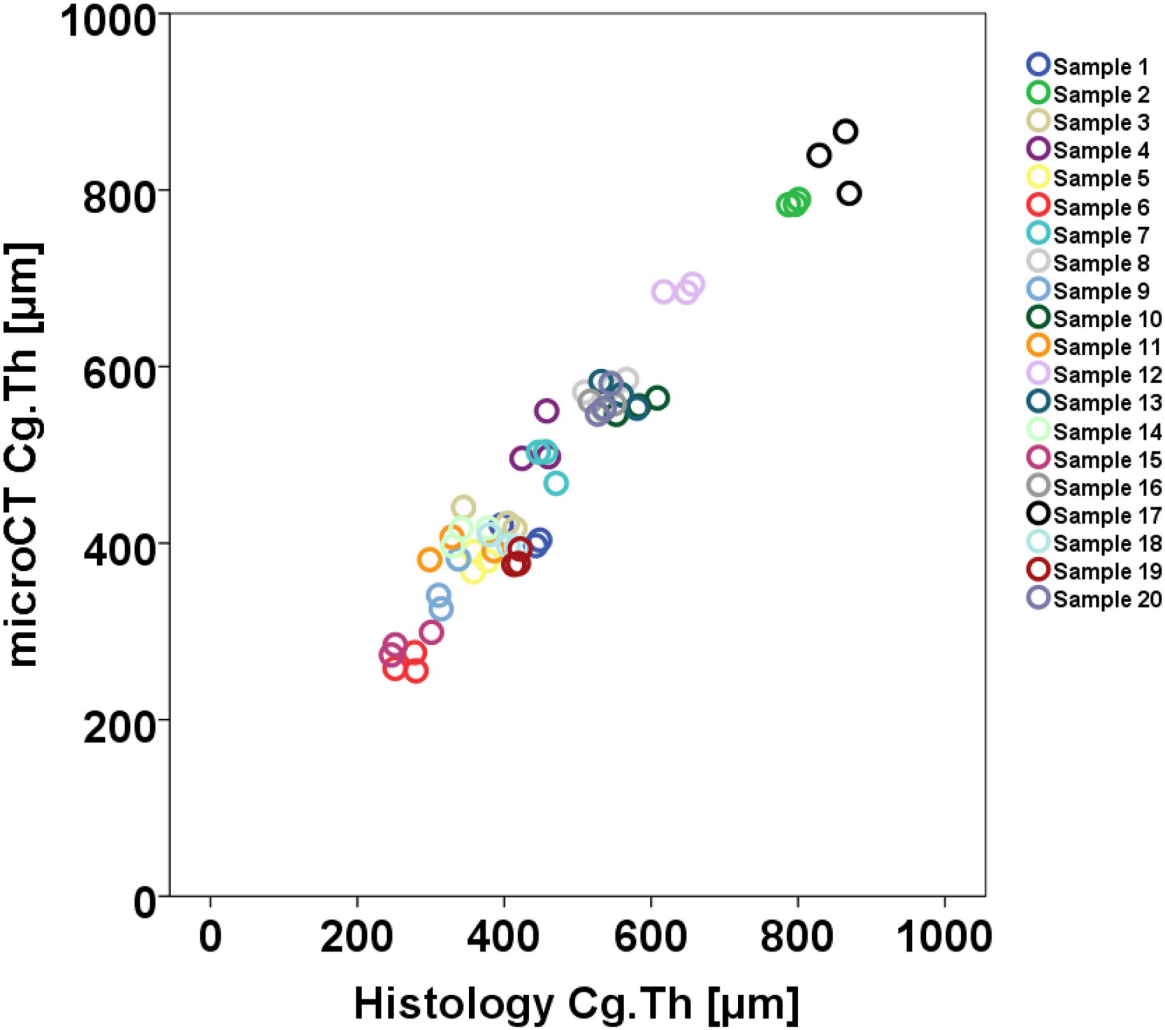
User reproducibility for cartilage segmentation. Plot of 2D cartilage thickness (Cg.Th) measured by three users segmenting a random selection of matching microCT and histology images; 10 femur and 10 tibia image pairs from both NO and OP joints, demonstrating user independence of the segmentation procedure. ICC (microCT) = 0.996 (0.992, 0.998); ICC (histology) = 0.993 (0.984, 0.997), p < 0.001.

Matching histology-microCT pairs analysed for 2D Cg.Th and muGr values revealed a significant correlation between the two methods in measuring 2D Cg.Th (R = 0.94), Fig 2a, and with no significant bias (Fig 2b). However, muGr showed no significant correlation (Fig 2c), and a strong proportional bias (Fig 2d). On average, cartilage measured with microCT was 39 μm higher compared to histology. High reproducibility was obtained for three users segmenting a random subselection of matching microCT and histology images; ICC (lower 95% CI, upper 95% CI) = 0.996 (0.992, 0.998) and 0.993 (0.984, 0.997), respectively (Fig 3).

### 3D QMA: Cartilage

**Fig 4:**
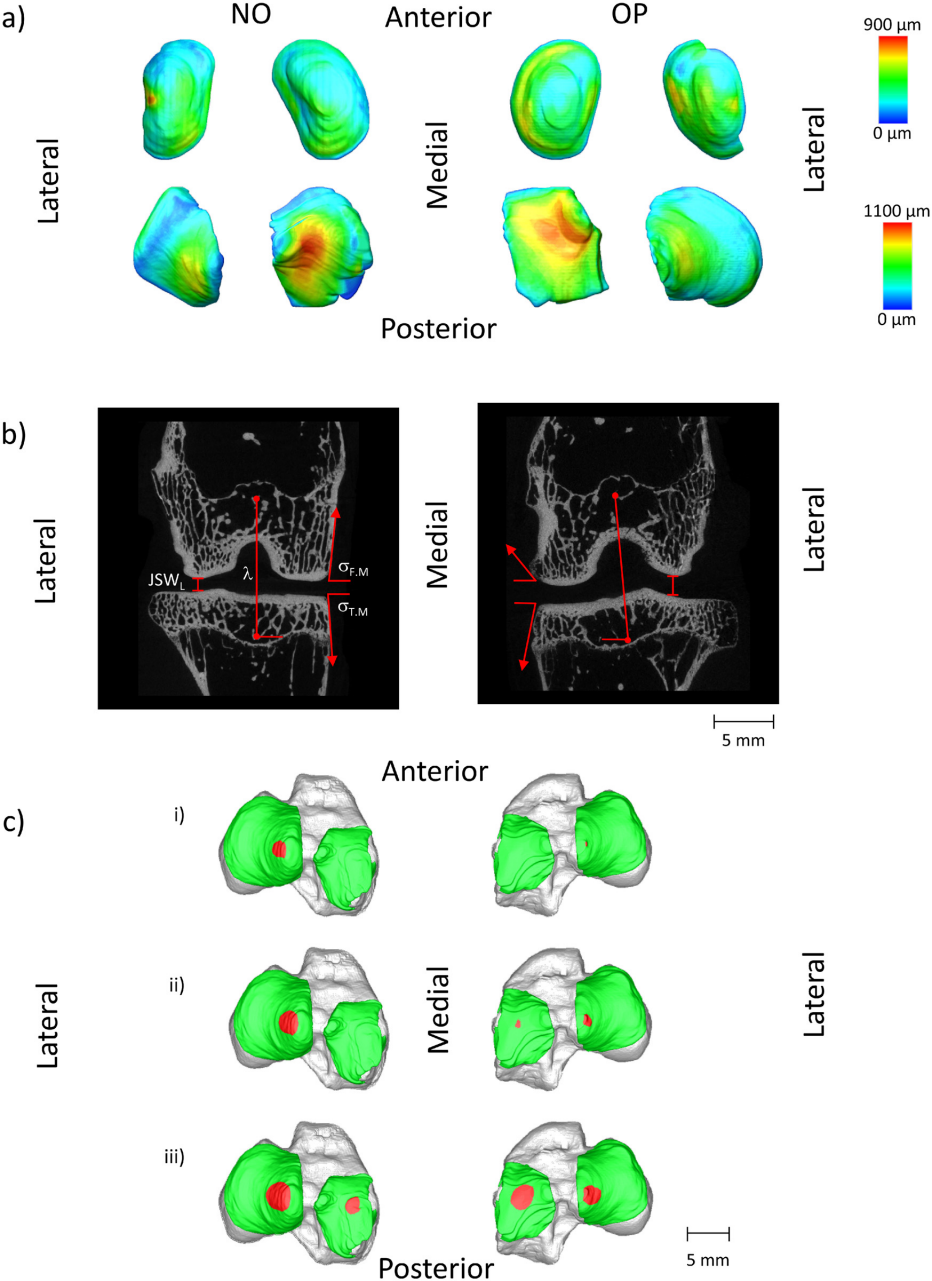
Typical examples of 3D QMA showing differences between OP and NO joints. (a) Thickness maps of top: femoral, and bottom: tibial cartilage compartments indicating increased Cg.Th in OP medial and lateral femoral compartments, and medial tibial compartment, as well as increased Cg.S and Cg.V in OP medial femoral condyle, and increased Cg.S in lateral tibial plateau. (b) Larger JSW, smaller α angle, and more acute medial σ angles are evident in the OP joint. (c) Upon virtual loading of the femur onto the tibia in a stepwise manner (i) the distance to reach first contact, χ - indicated in red—is significantly higher in lateral OP compartments compared to NO. (ii) and (iii) Once contact is reached, rate of increase, *m*, rises significantly faster in medial OP compartments.

Morphometric measurements of cartilage alone, two bones (tibia and femur) and *in situ* joint were obtained (Tables 1 and 2) and compared between OP and NO samples. 3D Cg.Th was, on average, significantly thicker in OP medial and lateral femoral condyles and medial tibial plateaus (Table 1 and Fig 4a). Medial femoral condyles also showed significantly more (~30%) surface and volume in OP samples. Lateral tibial plateaus only showed a significantly higher Cg.S (~35%) in OP samples. No significant differences were observed in Cg.S/Cg.V.

**Table 1:** 3D QMA Results for cartilage, bone and 2D OARSI, histology and microCT scores.

	Femur					Tibia				
	NO		OP			NO		OP		
	mean	± SD	mean	± SD	p	mean	± SD	mean	± SD	p
**Cartilage**										
Cg.Th (μm)										
Lateral	426	53	495	43	**	496	68	498	39	
Medial	380	27	432	78	*	688	59	720	62	*
Cg.S (mm^2^)										
Lateral	132	33	125	23		103	30	139	36	*
Medial	104	20	135	37	*	132	36	131	36	
Cg.V (mm^3^)										
Lateral	25.5	5.9	24.3	5.9		20.8	6.9	25.3	6.0	
Medial	18.6	4.6	24.2	9.6	*	23.5	6.6	23.1	6.7	
Cg.S/Cg.V (mm^-1^)										
Lateral	5.2	0.6	5.3	1.1		5.2	1.1	5.5	0.9	
Medial	5.7	1.3	5.8	0.8		5.7	0.9	5.8	1.0	
**Bone Margins**										
σ (°)										
Lateral	61.6	3.1	61.1	2.7		74.4	6.4	72.5	5.5	
Medial	72.4	8.3	57.5	9.0	**	87.0	5.3	77.6	5.4	**
ρ (°)	-5.11	0.92	-5.49	0.78		-		-		
**Cortical Bone**										
Ct.Th (μm)										
Lateral	733	37	714	29	*	691	57	694	99	
Medial	690	49	705	93		813	63	838	97	
Ct.Po (%)										
Lateral	16.0	2.7	16.4	1.8		19.3	1.5	19.8	2.1	
Medial	17.3	3.1	17.3	4.3		17.5	1.8	18.4	2.4	
**Epiphyseal Bone**										
BS (mm^2^)										
Lateral	1231	218	1160	262		331	91	303	115	
Medial	1230	238	1188	190		757	215	608	281	
BV (mm^3^)										
Lateral	161	35	137	40	*	60	11	44	8	**
Medial	171	38	139	19	*	101	29	70	30	**
BS/BV (mm^-1^)										
Lateral	7.72	0.55	8.63	0.94	**	5.57	1.29	7.33	1.49	**
Medial	7.25	0.52	8.82	2.60		7.10	1.27	8.69	1.13	**
BV/TV (%)										
Lateral	47.4	3.5	44.6	4.1		60.6	8.5	53.4	8.1	*
Medial	47.4	4.4	42.4	8.7		46.4	5.3	37.4	7.2	**
Tb.Th (mm)										
Lateral	0.34	0.04	0.31	0.05		0.49	0.10	0.38	0.07	**
Medial	0.35	0.04	0.31	0.05	*	0.43	0.08	0.37	0.06	**
Tb.Sp (mm)										
Lateral	0.57	0.05	0.60	0.06		0.40	0.05	0.43	0.06	
Medial	0.77	0.37	0.68	0.12		0.53	0.05	0.69	0.26	
Tb.N (mm^-1^)										
Lateral	1.59	0.10	1.57	0.12		1.89	0.14	1.91	0.23	
Medial	1.36	0.26	1.42	0.17		1.62	0.16	1.44	0.34	
Conn.D (mm^-3^)										
Lateral	4.10	0.41	4.80	0.69	**	3.97	0.80	4.38	1.00	
Medial	3.67	0.44	4.47	1.04	*	5.49	0.83	5.18	1.15	
DA (1)										
Lateral	1.65	0.10	1.57	0.10		1.46	0.06	1.40	0.08	
Medial	1.53	0.07	1.50	0.07		1.38	0.05	1.37	0.09	
**OARSI score**										
Safranin O—fast green staining	2.1	0.9	4.0	0.8	**	2.2	1.0	3.8	1.2	**
Structure	1.3	0.7	2.3	1.3	*	2.0	0.9	4.9	3.2	**
Chondrocyte density	0.1	0.3	0.6	0.8		0.4	0.5	1.4	1.2	**
Cluster formation	0.1	0.2	2.0	1.3	**	0.4	0.3	1.3	0.7	**
OARSI total	3.6	1.4	9.0	3.2	**	4.9	1.6	11.4	4.8	**
**Histology Score**										
Osteophytes	0.0	0.0	0.6	0.7	**	0.2	0.6	2.2	1.0	**
Tidemark breach	0.1	0.3	1.4	1.1	**	0.1	0.1	0.4	0.7	
Histology total	3.7	1.4	10.9	4.2	**	5.6	2.4	13.9	6.0	**
**CT Score**										
Surface structure	1.2	0.7	1.8	0.6		0.9	0.8	1.5	1.0	
Cartilage thickness	0.6	0.4	1.5	0.9	**	0.6	0.7	0.8	0.8	
Osteophyte size	0.1	0.4	0.8	0.4	**	0.4	1.1	2.4	1.1	**
Osteophyte shade	0.1	0.2	0.3	0.3	*	0.0	0.0	0.8	0.3	**
Bone remodelling	0.2	0.4	0.7	0.3	**	0.2	0.4	0.8	0.3	**
microCT total	2.3	1.7	5.2	1.7	**	2.0	2.4	6.1	3.1	**

Mean ± SD, where * and ** indicate significant differences between NO and OP (p < 0.05 and 0.01, respectively).

**Table 2:** 3D QMA Results for whole joint measures.

	NO		OP		
	mean	± SD	mean	± SD	p
JSW (mm)					
Lateral	1.1	0.3	1.9	0.6	**
Medial	1.0	0.1	1.2	0.3	*
λ (mm)	3.5	0.1	3.6	0.1	
α (°)	96.7	8.0	91.0	7.4	*
β (°)	94.4	3.4	93.7	3.2	
γ (°)	169.5	4.7	172.3	3.8	
τ (°)	-0.65	1.39	-0.15	3.44	
Χ (mm)					
Lateral	0.15	0.05	0.60	0.35	**
Medial	0.23	0.12	0.30	0.19	
*m* (mm^2^/mm)					
Lateral	38.2	8.2	71.0	40.0	**
Medial	49.9	12.8	45.4	11.1	

Mean ± SD, where * and ** indicate significant differences between NO and OP (p < 0.05 and 0.01, respectively).

### 3D QMA: Bone

Detection of osteophytes was measured using σangles, where reduced (more acute) angles indicated the presence and increased size of osteophytes. In OP medial compartments compared to NO joints from both femurs and tibias, σ was significantly reduced by 21% and 11%, respectively (Table 1 and Fig 4b), while no significant differences were seen laterally. Similarly no difference was observed for ρ; a measure of altered femoral condylar structure. In subchondral cortical bone, only a slight—albeit significant—reduction of 20 μm in cortical thickness of the OP lateral femoral condyle was seen. In tibial medial and lateral aspects of subchondral epiphyseal bone, thinner trabeculae, reduced BV and BV/TV, and increased BS/BV were observed (Table 1). In femoral condyles, similar but not significant trends were observed, where Conn.D was significantly different in both compartments. No differences were observed in Tb.Sp, Tb.N or DA.

### 3D QMA: *in situ* joint

JSW was significantly larger in both lateral (1.9 ± 0.6 mm vs. 1.1 ± 0.3 mm) and medial (1.2 ± 0.3 mm vs. 1.0 ± 0.1 mm) aspects of OP compared to NO joints (Table 2 and Fig 4b). The vector linking the centre of mass of the tibia and femur, as a measure of altered joint mechanics between the two bones, indicated that neither vector length, λ, nor angles β and γ, were significantly different, however, α was reduced in OP (91°) compared to NO (97°) joints. Upon virtual loading of the femur onto the tibia in a stepwise manner (Fig 4c), in order to measure the altered contact in OP joints, the distance to reach first contact, χ, was significantly higher in lateral OP compartments. Once contact was reached, the rate at which contact area increased, *m*, rose significantly faster in medial OP compartments (Table 2).

### Scoring: Cartilage

OP samples had less Safranin-O staining, a perturbed cartilage structure, an altered chondrocyte density (tibia only), and an increased cell cluster formation. Osteophytes were observed in femurs and tibias of OP samples, yet tidemark breaching was only observed in femurs (Table 1). MicroCT scores showed a significantly thicker OP femoral cartilage, larger osteophytes with brighter shades of grey in OP femurs and tibias, and greater evidence of bone remodelling. Total scores (OARSI, histology and microCT) were significantly higher (2–3 times) in OP joints (Table 1).

### Bivariate Regressions: *in situ* joint

**Fig 5:**
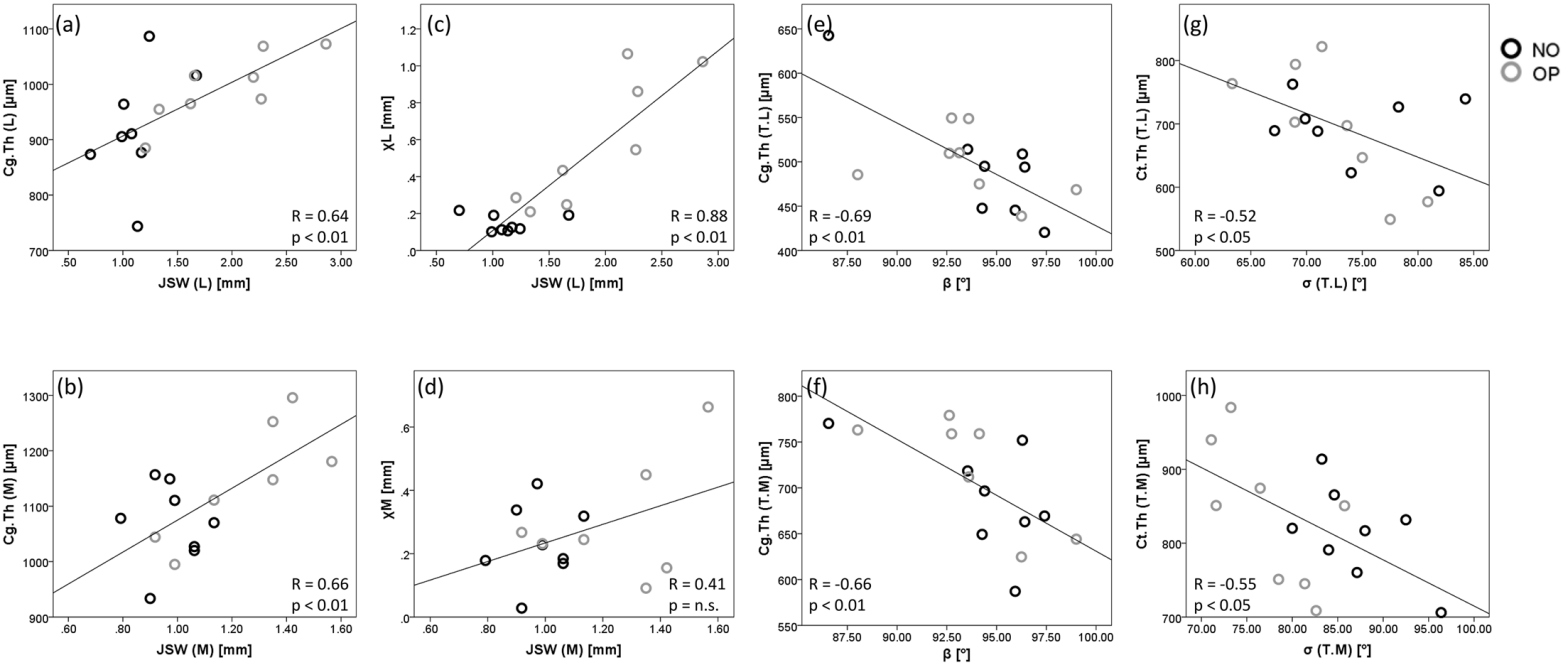
Bivariate Regressions of QMA measures. Relationships between QMA measures indicate significant associations between and within tissue measures (Cg.Th, Ct.Th) and whole joint measures (JSW, χ, β, σ). Correlations were observed between Cg.Th and JSW both (a) laterally (R = 0.64, p < 0.01) and (b) medially (R = 0.66, p < 0.01), where Cg.Th is the addition of mean tibial and femoral compartmental values. This was also seen laterally (c) but not medially (d) for JSW and χ (R = 0.88, p < 0.01). The β angle is strongly negatively correlated with both (e) lateral and (f) medial tibial Cg.Th (R = -0.69 and -0.66, respectively, p < 0.01), and Ct.Th is negatively correlated with σ in the (g) lateral and (h) medial tibia (R = -0.52 and -0.55, respectively, p < 0.05).

Associations were observed between Cg.Th and JSW both laterally (R = 0.64, p < 0.01) and medially (R = 0.66, p < 0.01), where Cg.Th represents the addition of mean tibial and femoral compartmental values (Fig 5a and 5b). Similar data were obtained for lateral but not medial JSW and χ (R = 0.88, p < 0.01 and 0.41, n.s, respectively), Fig 5c and 5d. Additionally, β angle (i.e. the angle λ made in antero-posteriorly) was negatively associated with both medial and lateral tibial Cg.Th (R = -0.69 and -0.66, respectively, p < 0.01) in both NO and OP (Fig 5e and 5f). A negative fit was seen between Cg.Th and BV/TV in lateral and medial femurs (R = -0.61 and -0.51, respectively, p < 0.05), while laterally, bone measures indicating thinner trabeculae, increased surface-to-volume, and lower density were associated with increasing JSW, χ and *m*. Joint twist angle, τ, was strongly associated with increasing rate of joint contact, *m*, laterally (R = 0.73, p < 0.01), and cortical thickness medially (R = 0.70, p < 0.01). Furthermore, Ct.Th was negatively associated with σ in the lateral and medial tibia (R = -0.52 and -0.55, respectively, p < 0.05), (Fig 5g and 5h).

**Fig 6:**
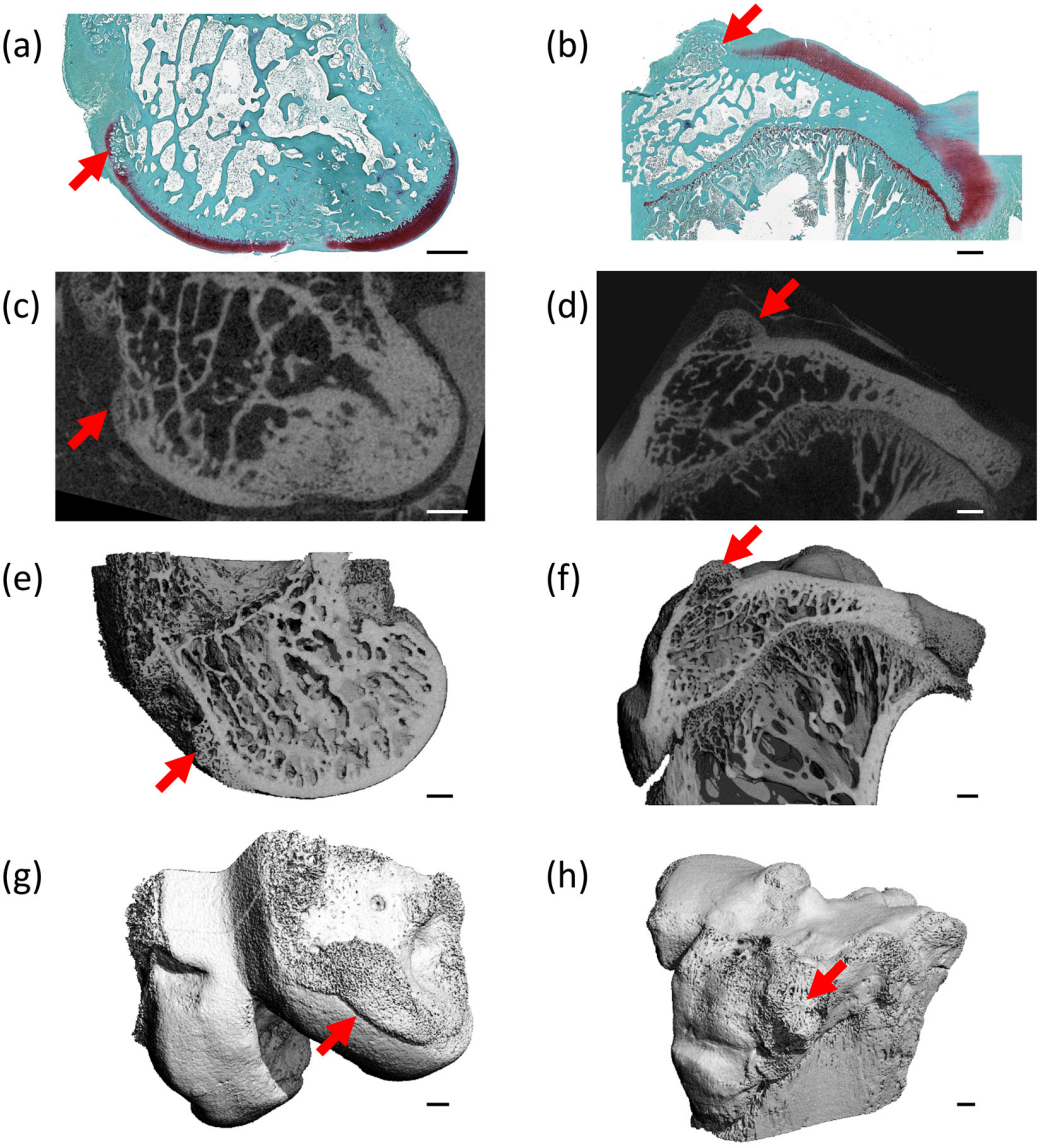
Visualisation of osteophytes. Osteophytes (red arrows) are indicated in the femur (a,c,e,g) and tibia (b,d,f,h) as visualised with (a-b) histology, (c-h) microCT: (c-d) the matching microCT image, (e-f) a cut-through the microCT 3D greyscale image showing low attenuating osteophyte masses, and (g-h) a 3D reconstruction of the microCT scan (PRE).

There was a strong correlation (R = 0.61–0.78, p < 0.05) between histology and 2D microCT scores (Table 3). Additionally, osteophyte shade (microCT scoring) was significant for σ_M_ in the femur (R = -0.58, p < 0.05) and tibia (R = -0.56, p < 0.05), osteophyte size (microCT scoring) for σ_M_ in the femur (R = -0.70, p < 0.01), and presence of osteophytes (histology) for σ_M_ in the tibia (R = -0.51, p < 0.05) (Table 3). This association of osteophyte measures between methods can be visualised in Fig 6. Interestingly, 2D Cg.Th did not correlate with either histology grade structure or the CT grade “thickness”.

**Table 3:** Bivariate linear regression results of the 3D QMA.

Parameters (Response—Predictor)	R		R^2^	m	b
Cg.Th (L)—JSW_L_	0.64	**	41%	97.08	809.65
Cg.Th (M)—JSW_M_	0.66	**	44%	288.08	786.79
χ_L_—JSW_L_	0.88	**	77%	0.49	-0.40
χ_M_—JSW_M_	0.41	ns	-	-	-
Cg.Th (T.L) -β	-0.69	**	48%	-11.60	1588.03
Cg.Th (T.M) -β	-0.66	**	44%	-12.20	1850.39
Cg.Th (F.L)—BV/TV (F.L)	-0.61	*	37%	-9.00	875.16
Cg.Th (F.M)—BV/TV (F.M)	-0.51	*	26%	-4.48	606.88
BV/TV (F.L)—JSW_L_	-0.66	**	44%	-4.38	52.72
BS/BV (F.L)—JSW_L_	0.82	**	68%	1.21	6.33
Tb.Th (F.L)—JSW_L_	-0.54	*	29%	-42.06	386.04
m_L_—BS/BV (F.L)	0.78	**	61%	29.15	-183.64
χ_L_—BS/BV (F.L)	0.70	**	50%	0.27	-1.81
τ- m_L_	0.73	**	54%	9.42	58.40
τ- Ct.Th (F.M)	0.70	**	50%	29.15	705.33
σ (T.L)—Ct.Th (T.L)	-0.52	*	27%	-0.04	100.29
σ (T.M)—Ct.Th (T.M)	-0.55	*	31%	-0.05	122.57

* and ** indicate significance (p < 0.05 and 0.01, respectively).

### Reproducibility of measurements

**Fig 7:**
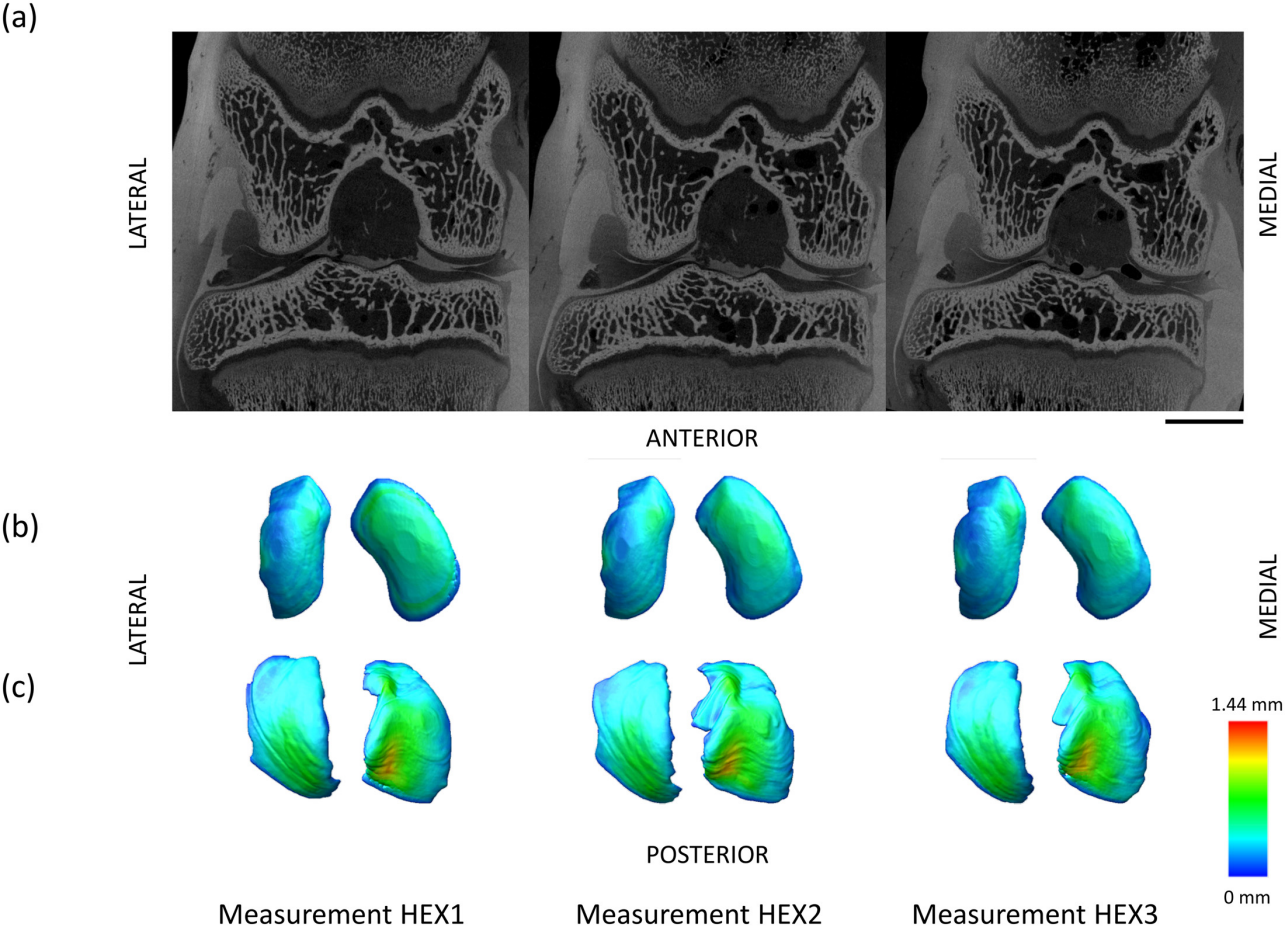
Measurement reproducibility. Typical HEX1/HEX2/HEX3 scans show excellent measurement reproducibility (ICC > 0.74) for cartilage, bone and *in situ* joint measures. (a) Bone morphometric ICC values were low for tibial BS/BV (0.077) and Tb.Th (0.403), due to penetration of Hexabrix^®^ into bone tissue. Scale bar = 5 mm. (b) Femoral and (c) tibial cartilage thickness maps demonstrate good reproducibility in cartilage compartments.

Excellent measurement reproducibility (ICC > 0.74) [36] was observed for cartilage, bone and joint measures, with ICC ranging from 0.754 (for χ_M_) up to 0.998 (for σ_T.M_) (Tables 4 and 5 and Fig 7). Bone morphometric ICC were unexpectedly low for tibial BS/BV (0.077) and Tb.Th (0.403), likely due to penetration of Hexabrix^®^ into bone tissue (Fig 7a). Precision errors, PE(SD) showed small absolute errors, and PE(%CV) were below 10% for all cartilage and whole joint measures, whereas Ct.Th values were above 15% (Tables 4 and 5).

**Table 4:** Intraclass correlation coefficients (ICC) and precision errors (PE) for cartilage and bone.

	Femur					Tibia				
	ICC	Lower 95%	Upper 95%	PE (SD)	PE (%CV)	ICC	Lower 95%	Upper 95%	PE (SD)	PE (%CV)
**Cartilage**										
Cg.Th (μm)										
Lateral	0.962	0.868	0.993	26.4	6.14%	0.990	0.964	0.998	8.1	1.50%
Medial	0.985	0.926	0.997	12.5	3.05%	0.961	0.863	0.993	24.9	4.08%
Cg.S (mm^2^)										
Lateral	0.930	0.731	0.987	7.77	7.15%	0.972	0.899	0.995	4.58	4.23%
Medial	0.906	0.673	0.982	8.09	6.72%	0.931	0.748	0.987	8.88	6.49%
Cg.V (mm^3^)										
Lateral	0.970	0.888	0.994	1.59	9.40%	0.983	0.937	0.997	0.83	3.94%
Medial	0.969	0.889	0.994	1.40	6.46%	0.967	0.886	0.994	2.17	7.11%
Cg.S/Cg.V (mm^-1^)										
Lateral	0.980	0.927	0.996	0.23	3.43%	0.982	0.933	0.997	0.07	1.47%
Medial	0.965	0.877	0.993	0.21	3.46%	0.952	0.828	0.991	0.19	3.94%
**Bone Margins**										
σ (°)										
Lateral	0.962	0.867	0.993	0.66	1.09%	0.987	0.952	0.998	1.38	2.22%
Medial	0.985	0.941	0.997	0.53	0.70%	0.998	0.993	1.000	0.45	0.52%
ρ (°)	0.995	0.984	0.999	0.06	1.28%	-	-	-	-	-
**Cortical Bone**										
Ct.Th (μm)										
Lateral	0.755	0.128	0.953	322	21.98%	0.804	0.271	0.963	187	15.30%
Medial	0.749	0.076	0.952	301	20.65%	0.786	0.278	0.958	334	18.81%
Ct.Po (%)										
Lateral	0.857	0.397	0.974	2.54	25.66%	0.898	0.621	0.981	1.58	21.87%
Medial	0.887	0.406	0.980	2.03	22.69%	0.911	0.686	0.983	1.44	26.29%
**Epiphyseal Bone**										
BS/BV (mm^-1^)										
Lateral	0.901	0.515	0.982	0.87	12.65%	0.077	-3.882	0.842	0.44	5.23%
Medial	0.834	0.316	0.969	0.83	11.14%	0.599	-0.227	0.920	0.61	7.29%
BV/TV (%)										
Lateral	0.918	0.650	0.985	3.69	6.94%	0.931	0.749	0.987	1.70	3.53%
Medial	0.930	0.683	0.987	3.25	6.19%	0.821	0.388	0.966	2.56	5.78%
Tb.Th (mm)										
Lateral	0.871	0.551	0.975	0.08	14.35%	0.403	-1.757	0.895	0.02	5.70%
Medial	0.832	0.411	0.968	0.05	12.59%	0.753	0.180	0.953	0.02	7.02%
Tb.Sp (mm)										
Lateral	0.985	0.947	0.997	0.01	3.01%	0.974	0.897	0.995	0.02	4.03%
Medial	0.992	0.972	0.998	0.01	2.48%	0.923	0.723	0.986	0.03	5.13%
Tb.N (mm^-1^)										
Lateral	0.983	0.940	0.997	0.05	2.58%	0.937	0.763	0.988	0.07	3.53%
Medial	0.991	0.967	0.998	0.05	2.42%	0.905	0.665	0.982	0.07	4.39%
Conn.D (mm^-3^)										
Lateral	0.946	0.481	0.991	0.35	5.83%	0.890	0.616	0.979	0.52	7.75%
Medial	0.947	0.778	0.990	0.40	6.37%	0.764	0.225	0.954	0.61	11.54%
DA (1)										
Lateral	0.944	0.788	0.990	0.05	3.40%	0.964	0.873	0.993	0.02	1.84%
Medial	0.942	0.760	0.989	0.04	2.85%	0.944	0.790	0.989	0.02	1.74%

Results expressed in absolute and percentage of 3D QMA parameters.

**Table 5:** Intraclass correlation coefficients (ICC) and precision errors (PE) for whole joint parameters.

	ICC	Lower 95%	Upper 95%	PE (SD)	PE (%CV)
JSW (mm)					
Lateral	0.942	0.779	0.989	0.07	9.68%
Medial	0.979	0.924	0.996	0.05	9.88%
Χ (μm)					
Lateral	0.888	0.547	0.979	0.04	9.58%
Medial	0.754	0.202	0.951	0.03	8.72%
*m* (mm^2^/mm)					
Lateral	0.980	0.928	0.996	1.11	5.26%
Medial	0.903	0.642	0.982	0.95	4.18%
λ (mm)	0.902	0.633	0.982	0.05	1.49%
α (°)	0.978	0.915	0.996	1.80	1.77%
β (°)	0.913	0.681	0.984	1.16	1.20%
γ (°)	0.981	0.931	0.996	1.46	0.92%
τ (°)	0.923	0.731	0.985	0.92	100.92%

Results expressed in absolute and percentage of 3D QMA parameters.

## Discussion

In this study, we established a novel 3D QMA that can distinguish between intact operated and non-operated joints in a rabbit model of OA. New metrics were defined and tested, including an angle to quantitatively measure osteophytes (σ), an angle to indicate erosion between the lateral and medial femoral condyles (ρ), a vector defining altered angulation (λ, α, β, γ) and a twist angle (τ) measuring instability and tissue degeneration between the femur and tibia, a length measure of joint space width (JSW), and a slope and intercept (*m*, Χ) of joint contact to demonstrate altered loading with disease progression. We demonstrated a correlation between microCT and histology and showed robust reproducibility of the measurements. Our study provides a novel approach to quantify macro and micro tissue measures in the *in situ* joint thereby presenting a valuable imaging and analysis tool in a medium-size preclinical animal model. Furthermore the image analysis, QMA, can be directly transferred to *in vivo* and longitudinal datasets.

The processing protocol to produce 3D microCT datasets of the preclinical OA model was directly compared with histomorphometry to assess measurement fidelity. Results showed the same 2D Cg.Th, with a small offset of 39 μm (~2 voxels), likely due to dehydration and shrinkage during histology processing, or partial-volume effect overestimating metrics in CT, as described previously [39, 40]. Earlier work has demonstrated a negative correlation between Safranin-O stain intensity and Hexabrix^®^ attenuation contrast in disarticulated joint cartilage, where increased GAG gave low contrast, and loss of GAG allowed influx of the contrast agent into the cartilage tissue [17]. Our data with intact joints failed to demonstrate such a correlation, indicating possible differential Hexabrix^**®**^ uptake in disarticulated relative to intact joints, better discrimination of small differences by histology compared to microCT, or large variation in Safranin-O staining across histology batches.

The 3D QMA proved sensitive to differences in comparing OP and NO rabbit joints. It is important to note that the use of the contralateral joint as control was not ideal, as changes in gait due to ACLT resection may have induced changes to the contralateral limb. The results demonstrated morphologic differences between the two joints, as well as mechanical alignment alterations between medial and lateral compartments. Larger JSW was observed in both lateral and medial OP samples compared to NO. Concomitantly, cartilage was thicker. These results are in line with previous work showing increased JSW in the lateral compartment [41] and cartilage thickness in femoral condyles [42] in early stages of disease in a rabbit trauma model of OA. It has been suggested that this occurs in the weight-bearing regions of the cartilage as a first response to the induced trauma [41, 43].

Upon virtual loading of the femur onto the tibia, OP took longer (higher χ) to make contact laterally. However, once contact was made, contact area increased rapidly with increasing step size. For this to occur, the two contacting surfaces (F.L and T.L) would need to be more conforming, have greater fibrillation (i.e. contact points), or have a medial femoral (or lateral tibial) shift creating earlier contact at the intercondylar notch. Previously described increased fibrillation in the lateral tibial cartilage in the rabbit ACLT model [44] is in line with higher Cg.S. observed in this work. There was no obvious indication of a tibio-femoral shift in QMA parameters: λ, α, β, γalthough this is reported in humans with ACL injury or deficiency [45, 46]. However, Fig 4a shows higher Cg.Th for the lateral femoral condyle near the intercondylar notch which could cause earlier contact.

Bone morphometry results in the OP joint indicated a reduction in BV concomitant with thinner trabeculae, and is consistent with previous work [47]. The lateral femoral condyle also had a thinner subchondral cortex and increased epiphyseal connectivity, consistent with other histomorphometry and microCT studies of ACLT effects in rabbit and canine models [48–51]. Additionally, in this work BV/TV in femoral condyles (lateral and medial) was negatively associated with Cg.Th, as shown previously in the rat [52]. Evidence in literature demonstrates that altered loading in the ACLT model can be directly linked to altered bone morphometry [48–50]. In concert with cartilage results, higher Cg.Th values would allow dissipation of applied load, resulting in less concentrated stresses and reduced bone volume [53, 54].

Considering the whole joint, although λ and ρ were not significantly different between NO and OP, α angle was significantly lower in the OP joint indicating additional mass (osteophyte growth) on the medial femur rather than a varus-valgus tilt or tibio-femoral shift, consistent with Batiste *et al* [41]. Higher BV and reduced medial femoral σ angle (measuring the presence of osteophytes) confirmed this finding. There was also a significantly higher medial tibia σ, but visual inspection indicated osteophyte volumes were much smaller in the tibia compared to the femur (Fig 6g and 6h). In fact, the results showed that measures for osteophytes were highly correlated for all methods (histology, microCT and 3D QMA) demonstrating that σ can be used to reproducibly and sensitively detect the presence of osteophytes.

Synovial joints are involved in both load-bearing and motion, thus we surmise that whole joint measures can inform not only structural change in tissues with OA disease, but also mechanical change [55]. Previous studies [56, 57] indicate that joint flexion influences cartilage thickness during the phases of gait. Specifically, when flexion angle was higher (low β), tibial Cg.Th was higher, and with higher extension (high β). However, it should be noted that the range of flexion-extension was designed to be consistent (~160°) and was therefore quite narrow (< 15°). Furthermore, twist angle, τ, was higher when *m*_L_ was higher, implying that when the tibia and femur were not physiologically aligned, they needed to be virtually “twisted” to an ideal orientation. The magnitude of τ was concomitant with a more rapid rate of contact laterally, likely due to contact at the intercondylar notch rather than directly on the plateau itself. Increase of τ with Ct.Th in the femoral condyle supports the altered loading scenario. Further investigation using motion analysis is required to link QMA parameters to altered joint loading and motion, as was performed previously for healthy rabbit motion [58].

As mentioned, a limitation of the study was the use of the contralateral limb as control. This inherently induces biomechanical changes. However, in view of the goal of developing sensitive metrics rather than describing the model, adherence to a policy of reducing animal numbers was considered more appropriate. However, the effect on the contralateral joint is still of interest for a trauma-induced model.

A further limitation was control of sample positioning. In this work, a B-spline interpolation was used to correct the 3D datasets to a consistent position; however this introduces some interpolation errors [34]. An improved approach would be a standardised positioning holder that allows scan-rescan in the case of multiple scans of the same sample (e.g. PRE, SIO as presented here) or future longitudinal monitoring approaches. This would improve reproducibility, as well as precision, power discrimination and consequently require fewer animals. This study shows that the QMA was sensitive to discriminate changes in an ACLT OA rabbit model with good reproducibility for most parameters, in line with previous microCT animal studies [38, 41, 59]. Immersion in Hexabrix^®^ significantly affected some bone parameters (Ct.Th, BS/BV and Tb.Th) due to contrast filling cortical pores and neighbouring trabeculae (Fig 7). Therefore, bone morphometry should be calculated from PRE or SIO scans where no contrast is included in the bone volumes of interest, as performed for the sensitivity analysis in this work.

The *in situ* rather than the *in vivo* nature of the method described currently represent a further limitation to monitor longitudinal changes within a single animal. 3R considerations are of increasing consequence in medical research, and the work presented here has potential to support these efforts. With additional development of non-toxic, cheaper contrast agents, longitudinal monitoring will reduce animal numbers further, and allow sensitive discrimination with the QMA developed here by following the time course of disease in one animal. The limitation will then be the number of scans (radiation exposure) per individual animal. An alternative would be a multimodal or standalone MRI approach, where any 3D dataset could be used to gather the described metrics (except bone morphometry).

## Conclusions

This study provides a novel 3D QMA to quantify macro and micro tissue measures in the joint of a rabbit OA model. New metrics were established consisting of: an angle to quantitatively measure osteophytes (σ), an angle to indicate erosion between the lateral and medial femoral condyles (ρ), a vector defining altered angulation (λ, α, β, γ) and a twist angle (τ) measuring instability and tissue degeneration between the femur and tibia, a length measure of joint space width (JSW), and a slope and intercept (*m*, Χ) of joint contact to demonstrate altered loading with disease progression, as well as traditional bone and cartilage and histo-morphometry measures. All measures, except cartilage measures, can be taken from an imaging protocol without contrast. For cartilage measures, a contrast agent allowing definition of the cartilage boundaries is required. Traditional measures were consistent with previous reports on the ACLT rabbit model, and support the reliability of the new measures. We demonstrate correlation of microCT and histology, sensitive discrimination of OA change and robust reproducibility.

## Abbreviations

2D: two-dimensional
3D: three-dimensional
α: angle in the XZ plane, of the vector connecting the centre of mass of the tibia to the femur
ACLT: anterior cruciate ligament transection
β: angle in the YZ plane, of the vector connecting the centre of mass of the tibia to the femur
BS: bone surface (mm^2^)
BS/BV: ratio of bone surface to bone volume (mm^-1^)
BV: bone volume (mm^3^)
BV/TV: ratio of bone volume to total volume (%)
Cg.S: cartilage surface (mm^2^)
Cg.S/Cg.V: ratio of cartilage surface to cartilage volume (mm^-1^)
Cg.Th: cartilage thickness, (calculated from 2D or 3D images) (μm)
Cg.V: cartilage volume (mm^3^)
CI: confidence interval
Conn.D: connectivity density (mm^-3^)
Ct.Po: cortical porosity (%)
Ct.Th: cortical thickness (μm)
DA: degree of anisotropy (1)
F: femur
γ: angle in the XY plane, of the vector connecting the centre of mass of the tibia to the femur
ICC: intraclass correlation coefficient
JSW: joint space width
L: lateral
λ: lambda
M: medial
*m*: rate of increase of contact area
microCT: micro computed tomography
MRI: magnetic resonance imaging
muGr: mean greyscale [–]
NO: contralateral control (non-operated)
OA: osteoarthritis
OARSI: OsteoArthritis Research Society International
OP: operated joint
PE(%CV): precision error (percent coefficient of variation)
PE(SD): precision error (standard deviation)
QMA: quantitative morphometric analysis
ρ: inclination angle of the line connecting the maxima of the medial and lateral femoral condyles
σ: angles measuring osteophytes on the lateral and medial edges of the femur and tibia
T: tibia
τ tau: angle of twist between the femur and tibia required to return them to an ideal/physiological orientation
Tb.N: trabecular number (mm^-1^)
Tb.Sp: trabecular spacing (mm)
Tb.Th: trabecular thickness (mm)
χ: distance travelled to first contact when virtually loading the femur onto the tibia
